# Effect of ACE mutations on blood ACE phenotype parameters

**DOI:** 10.1371/journal.pone.0308289

**Published:** 2024-10-08

**Authors:** Olga V. Kryukova, Dmitry O. Korostin, Vera A. Belova, Valery V. Cheranev, Zhanna A. Repinskaia, Igor V. Uporov, Steven M. Dudek, Olga A. Kost, Denis V. Rebrikov, Sergei M. Danilov

**Affiliations:** 1 Faculty of Chemistry, M.V. Lomonosov Moscow University, Moscow, Russia; 2 Center for Precision Genome Editing and Genetic Technologies for Biomedicine, Pirogov Russian National Research Medical University, Moscow, Russia; 3 Department of Medicine, Division of Pulmonary, Critical Care, Sleep and Allergy, University of Illinois at Chicago, IL, United States of America; Max Delbruck Centrum fur Molekulare Medizin Berlin Buch, GERMANY

## Abstract

**Background:**

Analysis of existing mutations of Angiotensin-I-Converting Enzyme (ACE) led us to hypothesize that the carriers of damaging ACE mutations (accompanied by low ACE levels) could be at risk for the development of late-onset Alzheimer’s disease (AD).

**Methodology/Principal findings:**

We quantified blood ACE levels in EDTA-containing plasma from 15 patients with 11 different heterozygous ACE mutations and estimated the effects of these mutations on ACE phenotypes, using a set of mAbs to ACE and two ACE substrates. We confirmed prior observations that the relatively frequent Y215C mutation in the N domain of ACE (present in ~1% of the population) is associated with both Alzheimer’s disease (AD) and reduced plasma levels of ACE (~50% of controls), indicating that it likely results in a transport-deficient protein. In addition, we identified another 4 mutations in both ACE domains (M118T, C734Y, V992M and V997M) which are also associated with decreased ACE levels in the blood, and, thus, could be putative risk factors for late-onset AD. One of these mutations, C734Y, is likely transport-deficient, while the other mutations appear to influence ACE catalytic properties. The precipitation of mutant M118T by mAb 2D1 and ACE mutant C734Y by mAb 3F10 increased 2-3-fold compared to native ACE, and therefore, these mAbs could be markers of these mutations. Also, we identified a mutation I989T, which is associated with increased ACE levels in the blood.

**Conclusions/Significance:**

Conducting a systematic analysis of blood ACE levels in patients with ACE mutations holds promise for identifying individuals with low blood ACE levels. Such individuals may be at increased risk for late-onset AD. The patients with transport-deficient ACE mutations may benefit from therapeutic treatment with a combination of chemical and pharmacological chaperones and proteasome inhibitors, as was demonstrated previously using a cell model of the transport-deficient ACE mutation, Q1069R [Danilov et al, PLoS One, 2010].

## Introduction

We hypothesized recently that the carriers of heterozygous loss-of-function (LoF) mutations of Angiotensin-I-Converting Enzyme (ACE) that are accompanied by low ACE levels may be at risk for the development of late-onset Alzheimer’s disease (AD). From a total of 1234 ACE mutations, 400+ of these mutations may be damaging based upon *in silico* predictions [1]. In prior work, blood ACE levels were measured directly in carriers of 20 mutations, while blood ACE levels were estimated indirectly in carriers of 42 other ACE mutations [2].

Numerous genes have been linked to AD risk, but AD etiology remains incompletely understood. Rare cases of early-onset familial AD are associated with mutations in amyloid precursor protein *APP* and presenilins (*PS1* and *PS2*) [3], while late-onset AD is multifactorial and associated with mutations in *APOE* and more than 30 [4] other different genetic risk loci involved in vascular dysfunction, immunity, inflammation, cholesterol metabolism, endocytosis, and ubiquitination.

One of these genes associated with AD encodes for Angiotensin-Converting Enzyme (ACE, CD143, EC 3.4.15.1) [4–6]. The combined frequency of >400 of these mutations that are predicted to be LoF-damaging ACE mutations in the general population is quite significant–up to 5% [1], which is comparable to the frequency of AD in the elderly population >70 y.o. Thus, the contribution of low ACE levels, associated with these damaging ACE mutations, may be underappreciated in the development of AD.

The mechanism of the association between “ACE gene–AD” could be direct and straightforward. ACE was shown to degrade the major component of the plaques, amyloid peptide, both *in vitro* [7] and in a human cell line transfected with human cDNAs encoding both amyloid precursor protein and ACE [8]. In particular, the N domain active center of ACE is considered to be responsible for the degradation of Aβ42 [9]. Thus, experimental data suggest that lower levels of ACE correspond to higher levels of Aβ42 and an increased risk of AD. However, in addition to causing low surface ACE expression, several other mechanisms have been proposed to explain the possible association of ACE mutations with AD [1].

The aim of this current work was to continue ACE phenotyping in carriers of different ACE mutations and to estimate the effect of these ACE mutations on different characteristics of the ACE phenotype, including catalytic properties. The final goal of the 1^st^ stage of the project “ACE-dependent AD” is to establish an Atlas of ACE mutations with maximally complete ACE phenotype parameters and their association with AD pathogenesis.

The most important (and clinically relevant) conclusion of this analysis will be the ability to identify real patients (and their families) with low ACE levels, and, particularly, those resulting from transport-deficient ACE mutations. The 2^nd^ stage of this project then would be to perform deep medico-genetic analysis of these families to reveal additional genetic factors in the development of AD in carriers of a given ACE mutation (i.e., “Personalized medicine in ACE-dependent AD”). Patients with transport-deficient ACE mutations and with the lowest ACE levels may benefit from preventive or therapeutic treatment with a combination of chemical and pharmacological chaperones and proteasome inhibitors to restore impaired surface ACE expression. This type of therapeutic intervention could be the 3^rd^ stage of the this project, namely, “Therapeutic delay of AD development”. The potential effectiveness of this approach has previously been reported using an *in vitro* model for another transport-deficient ACE mutation (Q1069R), which is causal for renal tubular dysgenesis-RTD [10].

## Materials and methods

### Study participants

The collection of human blood samples and the protocol for the whole exome screening of newborns was reviewed and approved by the Ethics Committee of the Kulakov National Medical Research Center for Obstetrics, Gynecology and Perinatology (protocol No. 9 from 10.22.2020). We used exome sequencing data from 637 healthy Russian donors previously processed in our laboratory as a control dataset. All corresponding procedures were carried out in accordance with institutional guidelines and the Code of Ethics of the World Medical Association (Declaration of Helsinki). The parents of the newborns gave written informed consent for the use of any data for scientific purposes. The whole study included EDTA-plasma samples from 15 newborns with 11 ACE mutations and 16 controls.

### Whole exome sequencing

Genomic DNA isolation, quality assessment, DNA library preparation, further enrichment with pre-capture sample pooling according to the “RSMU exome” protocol" and sequencing were performed as recently described [2, 11].

### Chemicals

ACE substrates, benzyloxycarbonyl-L-phenylalanyl-L-histidyl-L-leucine (ZPHL) and hippuryl-L-histidyl-L-leucine (HHL) were purchased from Bachem Bioscience Inc. (King of Prussia, PA) and Sigma (St. Louis, MO). Other reagents (unless otherwise indicated) were obtained from Sigma (St. Louis, MO).

### Antibodies

Antibodies to human ACE used in this study, which recognize native conformations of the N and C domains of human ACE, were described previously [12–14].

### ACE activity assay

ACE activity was measured using a fluorimetric assay with two ACE substrates, 2 mM ZPHL or 5 mM HHL [15, 16]. The parameter ZPHL/HHL ratio was calculated as the ratio of the rates of the hydrolysis of ZPHL and HHL by the definite ACE sample [17].

### Immunological characterization of the blood ACE

The amount of immunoreactive ACE protein in EDTA-plasma was quantified by a modified immunoassay, in which native ACE from plasma samples was captured by anti-ACE monoclonal antibodies (mAbs) recognizing conformational epitopes on the surface of ACE molecules. Microtiter (96-well) plates (Corning, Corning, NY) were coated with anti-ACE mAbs via goat anti-mouse IgG (Invitrogen, Rockford, IL or IMTEK, Moscow, Russia) bridge and incubated with plasma samples diluted 10 times. Next, after washing away the unbound ACE (together with EDTA and possible ACE inhibitors) precipitated ACE activity was quantified directly in the wells of microtiter plates fluorometrically with ZPHL or HHL as substrates [2, 15, 16]. Conformational fingerprinting of blood ACE was performed as described earlier using a set of mAbs to different epitopes of ACE [12, 13].

### Statistical analysis

Values of ACE activity with different substrates for each individual, as well as other parameters characterizing ACE phenotype, are expressed as means ± SD from at least 3 independent experiments with duplicates. Significance was analyzed using the Mann-Whitney test.

Predictions and scores which account for evolutionary conservation and structural features were performed using four predictive tools as described for S1 Table in S1 File in [1].

### Localization of AD-associated ACE mutations on ACE globule

Coordinates of X-ray model of the human somatic ACE for some figures were downloaded from the PDB accession #7Q3Y [18].

## Results and discussion

### Quantification of blood ACE in carriers of ACE mutations

Previously, we have established an approach for characterization of ACE in the blood (“blood ACE phenotyping”) which included the following parameters: measurement of ACE activity, quantification of immunoreactive ACE protein, and detection of conformational changes in blood ACE using a set of mAbs to different epitopes on the surface of the ACE globule [12, 13–15, 19]. Unfortunately, most sequencing facilities operate only with EDTA-containing plasma, which makes it impossible to measure ACE activity directly due to the extraction of zinc-ion from the active centers of the enzyme by a chelating agent. However, ACE levels in EDTA-containing plasma can be estimated by precipitation of ACE by mAbs with subsequent washing-out of EDTA, and then ACE activity can be measured. In a separate work, we have demonstrated that ACE levels detected with mAb 9B9 in EDTA-containing plasma was practically identical with ACE levels detected in serum from the same patients (coefficient correlation of ACE levels in EDTA-containing plasma and serum from the same 12 patients was 0.911 –Kryukova et al., 2024 paper in preparation). Moreover, the use of different mAbs to ACE together with the use of different ACE substrates allowed detailed characterization of ACE from EDTA-containing plasma of patients with 10 different ACE mutations in the N domain of the enzyme, the signal peptide, and the cytoplasmic tail [2].

**Fig 1:**
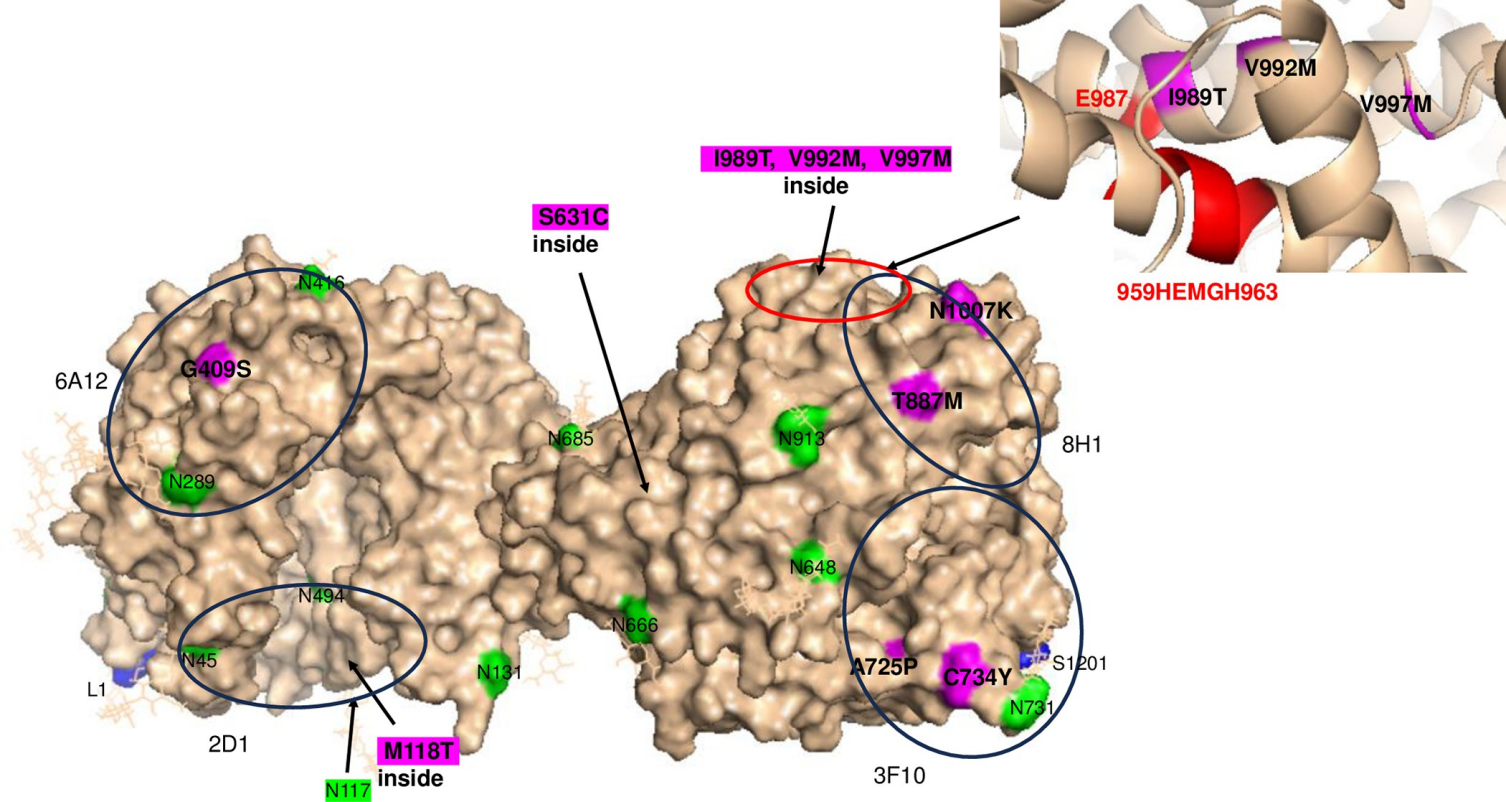
Localization of tested ACE mutations in the N and C domains of ACE. The positions of ACE mutations are shown on the Cryo-EM structure of the truncated (1–1201) human somatic ACE (PDB 7Q3Y) [18] using molecular surface representation. Key amino acids are denoted using somatic ACE numbering. The surface is colored light beige with specific amino acid residues color coded as follows: ACE mutations are highlighted in magenta and additionally marked by arrows; putative Asn glycosylation sites are highlighted in green; the first residue in the N-domain of somatic ACE is marked with its number L1, while the last visible residue in the C-terminal end of this truncated somatic ACE is marked with its number S1201. Both terminal residues are highlighted in blue. The epitopes for some mAbs to the N and C domains are shown as black circles for orientation with a diameter 30 Å, which corresponds to 700 Å^2^ of the area covered by this mAb. Insert: the mutations I989T, V992M and V997M are highlighted in magenta; active site residues are highlighted in red.

Here, we estimated plasma ACE levels in 15 newborn carriers of 11 other ACE mutations and in 15 newborn controls. One ACE mutation, p.Gln25Leu, appeared to be in the signal peptide (SP2), which is cleaved during maturation and could not be seen in mature protein. One patient, VKK056, was a carrier of two ACE mutations, namely, Y215C which was described earlier [2, 4, 16] and a T887M [6, 20]. Fig 1 provides an overall view of the locations of 10 of these ACE mutations marked with magenta color in a molecular model of mature two-domain somatic ACE [18]. The position of Y215C described earlier [2] is not seen in Fig 1, as it is on the opposite side of the N domain globule. Two new mutations appeared to be in the N domain, one of which is on the surface (G409S), and the other is inside the globule (M118T). Eight mutations were in the C domain. Four of these were found on the surface (A725P, C734Y, T887M and N1007K), and the other four were inside the globule (S631C, I989T, V992M and V997M) (Fig 1). The positions of mutations that could not be seen because they are located inside the globule are marked by arrows.

**Fig 2:**
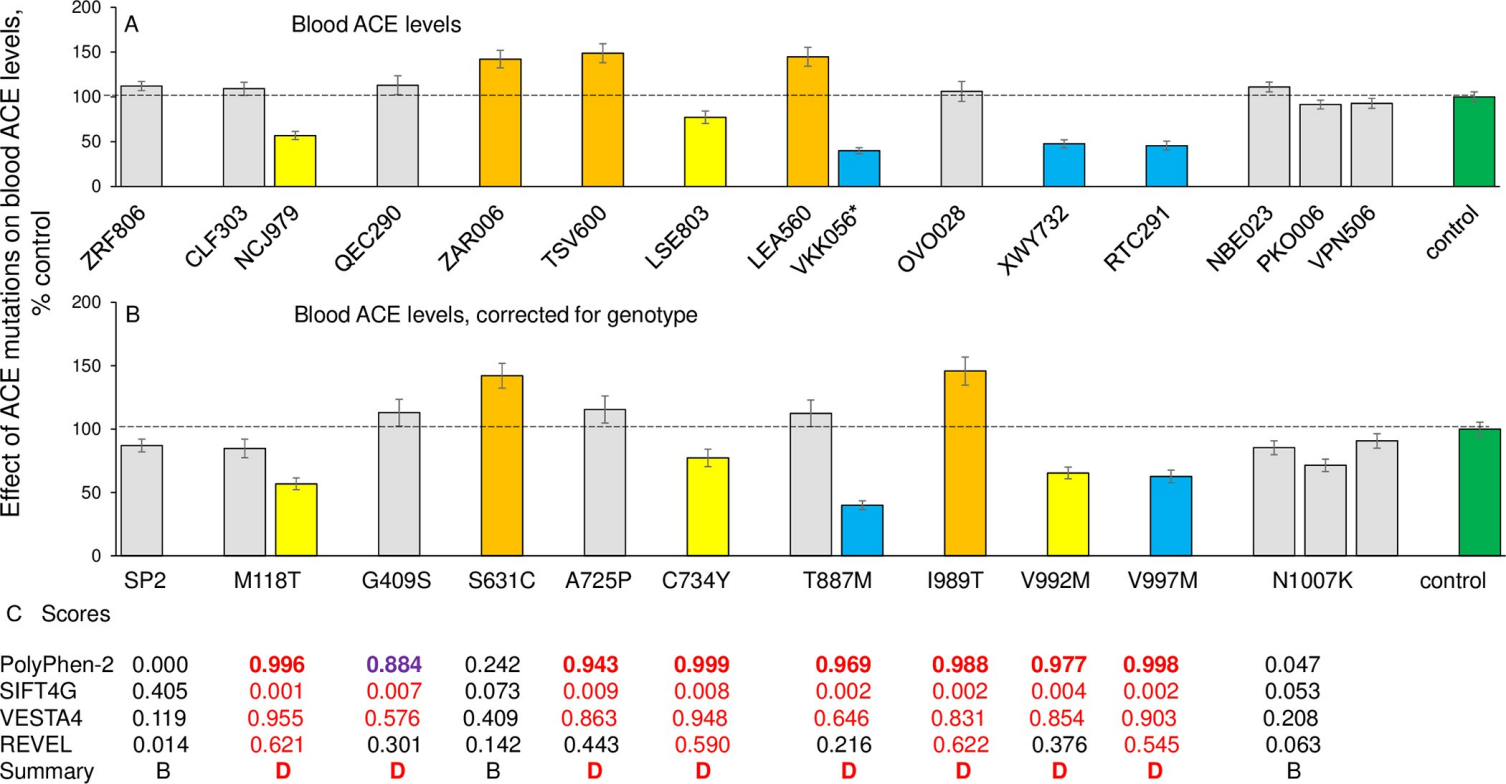
Quantification of blood ACE levels in carriers of ACE mutations. Blood ACE protein was precipitated from EDTA-plasma by mAb 9B9 (with epitope on the surface of the N domain of ACE), and its activity was quantified fluorometrically using ZPHL as a substrate. **A)** The immunoreactive ACE protein was quantified in plasma samples of 15 newborn carriers with 11 different ACE mutations. The asterisk indicates that ACE in the blood of patient VKK056 had two mutations, T887M and Y215C. **B)** ACE levels in plasma samples were adjusted according to the donor’s genotype I/D polymorphism [25, 26]. ACE levels in the blood of each person were presented as the mean of several experiments with standard deviation. **C**) Predictions of the potential damaging effects of 11 mutations on the ACE protein using 4 different predictive tools were derived from S1 Table in S1 File [1]. Values shown in red are predicted to be damaging by the listed predictive engine; values in black are predicted to be benign. Data were expressed as % of ACE levels from the corresponding value for control pooled plasma samples from donors without ACE mutations (green bars). Bars highlighted with orange and brown indicate samples with ACE levels higher than 120% and 150% of controls, respectively. Bars highlighted with yellow, and blue indicate samples with ACE levels lower than 80% and 50% of controls, respectively.

ACE levels in the EDTA-containing plasma samples of newborn carriers of 11 ACE mutations (determined with mAb 9B9) are shown in Fig 2A as a percentage of the mean blood ACE levels from 22 control samples without ACE mutations. However, an exact (quantitative) estimation of the effect of ACE mutations on blood ACE levels in a given individual requires knowing the ACE genotype on ACE I/D polymorphism, because both blood and tissue ACE levels are influenced by this ACE genotype [15, 21–23]. Thus, blood ACE levels in carriers of the DD genotype were reported to be 66% higher than in carriers of the II genotype [15, 24]. Therefore, ACE levels in the blood samples tested here were corrected according to the ACE genotype in a given individual, according to [25, 26]. The results are presented in Fig 2B, which demonstrates the quantitative influence of each mutation on blood ACE level.

First of all, we found that the plasma ACE level in patient VKK056, which expressed two ACE mutations (Y215C and T887M), was less than 50% of controls (Fig 2A and 2B). This result is consistent with prior observations that the relatively frequent (~1%) and AD-associated Y215C mutation in the N domain of ACE is damaging [2], and likely transport-deficient. In addition, we identified another ACE mutation in the N domain (M118T), and 3 mutations in the C domain (C734Y, V992M and V997M), which also had significantly reduced blood ACE levels (Fig 2B). Thus, these mutations also could be considered as candidates for transport-deficient mutations that may increase the risk of late-onset AD. ACE levels in the blood of carriers of another 7 ACE mutations were practically unchanged or even elevated (Fig 2A and 2B). In particular, a mutation in a signal peptide, p.Gln25Leu, did not influence ACE levels, similarly to the non-frameshifting insertion, p.L18_L19insPL, tested earlier [2], which is not associated with AD risk [27]. Two mutations located inside the C domain globule (S631C and I989T) resulted in increased ACE levels detected in the blood. Note that there was only one blood sample from each person for 8 of these mutations (Fig 2A), and therefore, the putative effects of these mutations (Fig 2B) on blood ACE levels should be considered as preliminary quantification. Nevertheless, we compared the efficacy of predictions of the damaging effects of 11 tested ACE mutations in ACE protein using 4 different predictive tools (Fig 2C), which were based on analyses of evolutionary, population genetic and protein 3D structural constraints. This comparison generally confirmed the accuracy of the predictions.

### Phenotyping of mutant ACEs using EDTA-plasma

In addition to damaging mutations leading to low surface ACE expression, such as Y215C or G325R [2], other ACE mutations may alter the inner structure of ACE and, therefore, change ACE catalytic activity. To identify putative carriers of such mutations, we applied here our previously established approach [12, 15, 16, 28–30], using precipitation of native ACE by mAbs to ACE from EDTA-plasma followed by the detection of enzymatic activity with two substrates, ZPHL and HHL, and calculation of ZPHL/HHL ratio of the rates of the hydrolysis of these substrates. Active centers located in the N and C domains of ACE hydrolyze these substrates with different efficiency. The N domain active center better hydrolyzes ZPHL, while the C domain active center better hydrolyzes HHL. Hence, not only the changes in ACE activity towards any of these substrates, but also the changes in the ratio of the rates of their hydrolysis (the ZPHL/HHL ratio), may indicate altered catalytic functions of one or another active site [17]. On the other hand, unchanged ZPHL/HHL ratio in combination with changed ACE levels in the blood may indicate changes in ACE expression.

**Fig 3:**
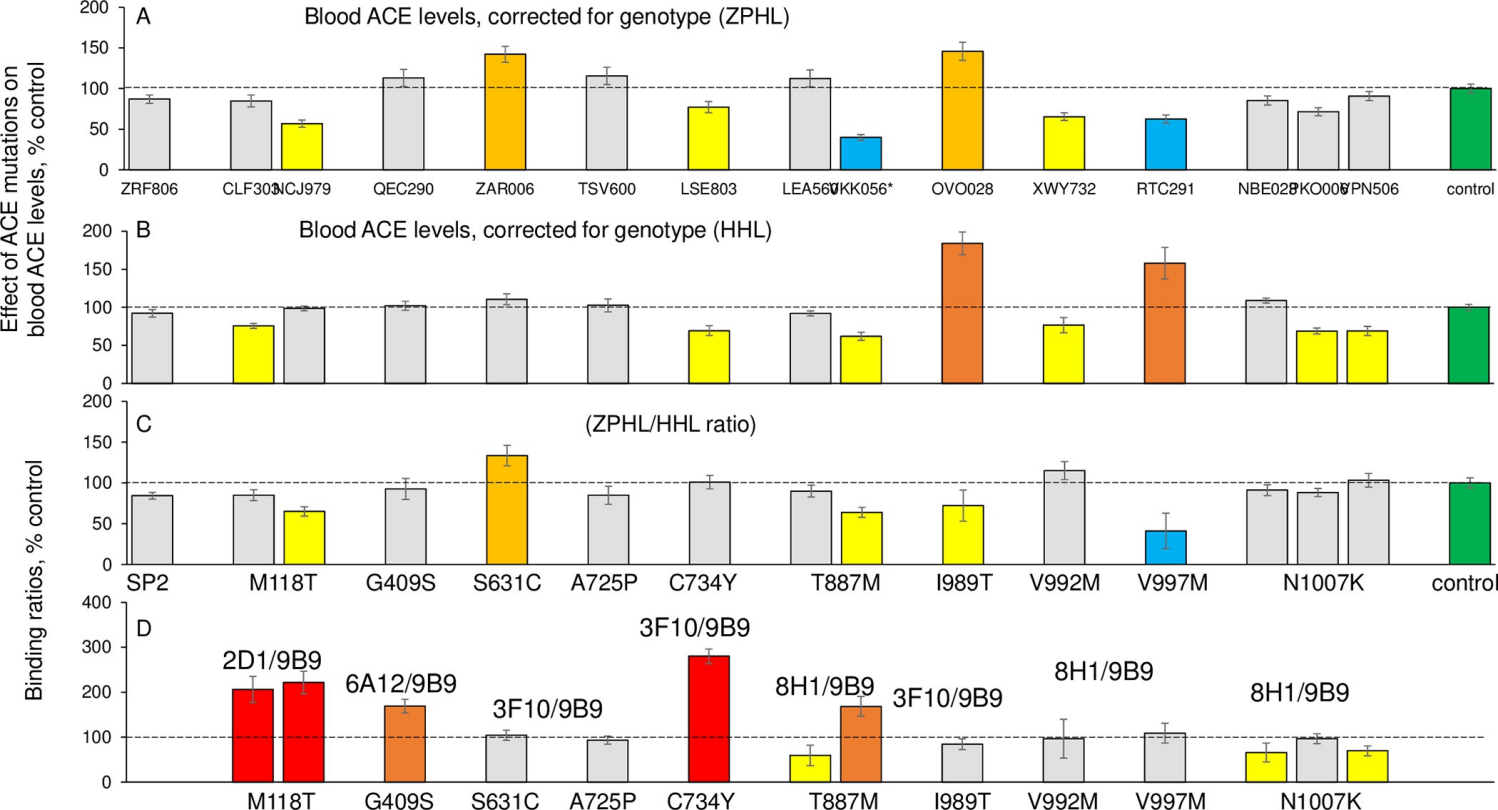
ACE phenotype for carriers of ACE mutations. **A-B.** Blood ACE protein was precipitated by mAb to the N domain (9B9), and precipitated ACE activity was quantified with substrate ZPHL (**A**) and HHL as in Fig 2. The ZPHL/HHL ratio was calculated for each carrier of the different ACE mutations (**B**). **C)** Precipitation of ACE protein from the blood of carriers of some ACE mutations using mAbs with various epitopes, including the region of these mutations normalized to the precipitation of ACE from the same sample by mAb 9B9. The coloring is the same as in Fig 2.

We compared the influence of 11 ACE mutations on apparent ACE levels in EDTA-plasma using different substrates, ZPHL and HHL, together with the ZPHL/HHL ratio. The level of ACE in the blood of patient VKK056 containing two mutations, Y215C and T887M, as determined with ZPHL or HHL, appeared to be decreased in both cases (Fig 3A and 3B), confirming the low expression of ACE. However, this putative transport-deficient mutation also induced some changes in the catalytic properties of ACE, as the ZPHL/HHL ratio was decreased (Fig 3C).

Among the other 4 ACE mutations with decreased ACE level as determined with ZPHL (Fig 2), the C734Y mutation on the surface of the C domain (Fig 1) could be considered as potentially transport-deficient because ACE level detected with both substrates was decreased, while the ZPHL/HHL ratio did not change (Fig 3C). Thus, this mutation may contribute to the risk factors of late-onset AD.

**Fig 4:**
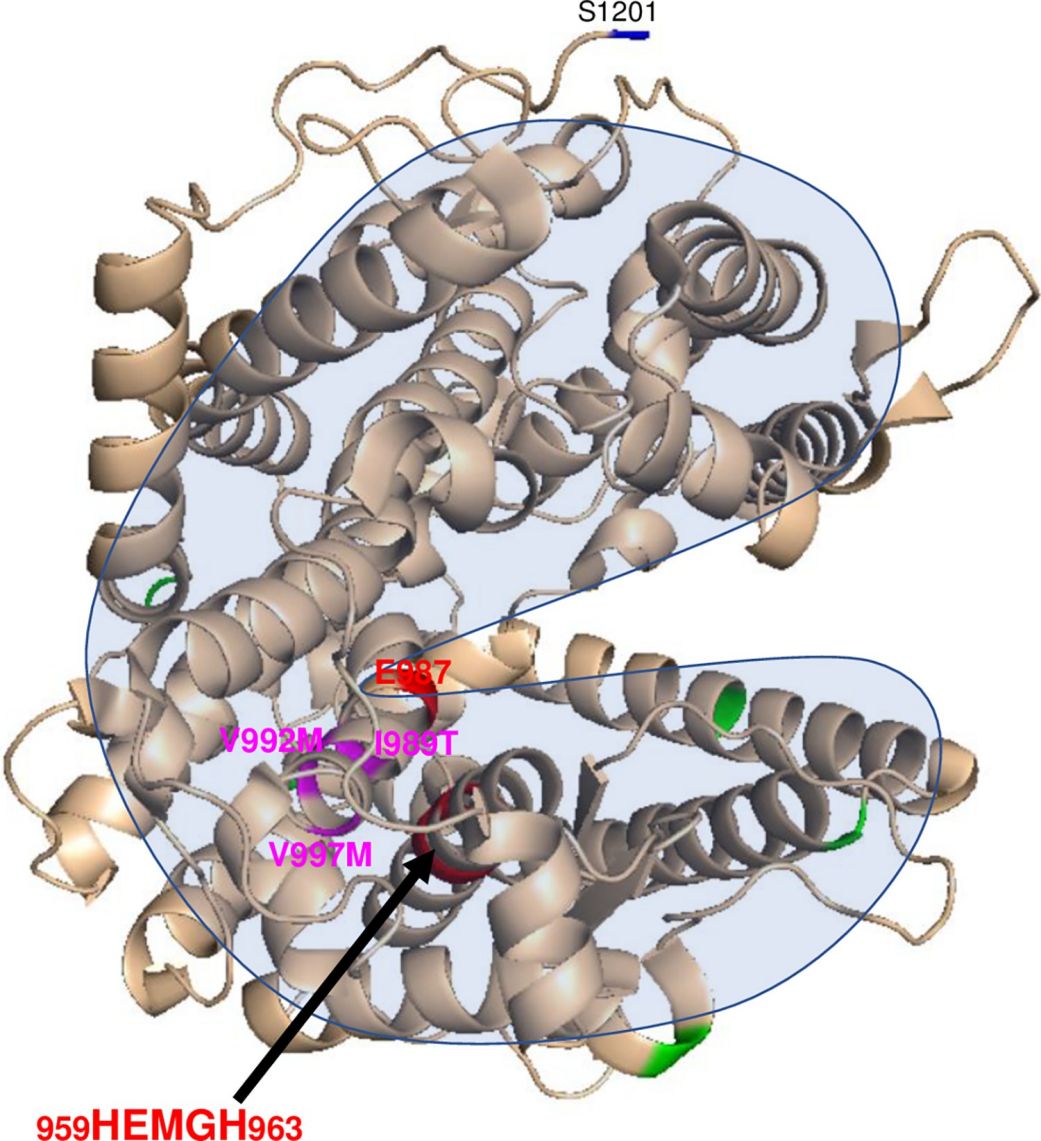
The position of the mutations I989T, V992M and V997M in the ACE C domain. The positions of ACE mutations are shown on the Cryo-EM structure of the truncated (1–1201) human somatic two-domain ACE (PDB 7Q3Y) [18] using ribbon representation. The coloring is the same as in Fig 1.

Another mutation, V997M, located deep inside the C domain globule (Fig 1) is unlikely to be transport-deficient, but it should be considered as a mutation directly changing the catalytic functions of ACE. While the ACE level in EDTA-plasma from the patient with this mutation was decreased as determined with ZPHL, the level determined with HHL appeared to be significantly increased (Fig 3B). As a result, the ZPHL/HHL ratio was remarkably decreased (Fig 3C). This amino acid residue 997 is located on a loop near the active site residues (Fig 1, insert, and Fig 4). Note that the distance between the imidazole ring of His959 (one of the active site residues) and the methyl group of Val997 was estimated to be 9.6 Å, which is a bit too far for a direct interaction. However, we could suggest possible mechanism of the influence of V997M mutation on ACE activity. An analysis of the effect of the V997M mutation was based on the protein structure and takes into account the interaction between the residue 997 and the His959 residue of the active site along the line connecting the side chains of these residues (S1 Fig in S1 File). The consequences of this mutation are considered that the methyl group of Val997.CG2 “disappears.” Note that the side chain of Met is longer (while Val is branched) and there would certainly be a change in the conformation of the amino acid residues surrounding residue 997 as a result of the mutation. Simultaneously, it is reasonable to allow the formation of an “empty” space where the side chain of Ala994 would shift. So, “in a straight line”, the residue 997 interacts with His959 through Ala994. The distance between the carbon atoms Ala994.CB and Val997.CG2 is 4.7 Å. Considering the size of the methyl groups, we can assume that they are in close contact and van der Waals and electrostatic interactions can exist between them. When the methyl group of Val residue is replaced with Met, the methyl group of Ala994 shifts towards residue 997 and occupies part of the vacated empty space in the globule. The distance from Ala994.CB to His959.ND1 is 5.2 Å, and to the hydrogen atom on the same nitrogen atom is 4.5 Å. Considering that the methyl group is a “proton” cloud with a radius of about 1.2 Å, there is definitely a contact between these groups, and Ala is involved to some extent in stabilizing the conformation of the His side chain. The shift of the methyl group of Ala994 towards the residue 997 would result to the disruption of the contact between residues 959 and 994, which, in turn, would affect the conformation of the His residue of the active site and ACE activity.

The mutation V992M is located in a nearby helix in proximity to the active site residues (Fig 1, insert) and resulted in decreased ACE activity (by less than 50%) determined with both ZPHL and HHL substrates, with an unchanged ZPHL/HHL ratio (Fig 3C). So, it is tempting to consider the mutation V992M as transport-deficient. Note that the side chain of the 992 residue is turned away from the active center, and thus, this mutation is unlikely to be directly influencing ACE catalysis. However, valine in this position is in a rather hydrophobic environment surrounded by Phe1109 and Ile989, positioned not farther than 5 Å. Thus, the substitution of hydrophobic Val for the longer and less hydrophobic Met may shift the chain within a rather dense protein structure so that this subtle change results in a slight decrease of ACE activity

The mutation I989T is located on the same helix just nearby (Fig 1, insert) and led to an opposite effect on ACE level detected with both ZPHL and HHL, together with decreased ZPHL/HHL (Fig 3C). Thus, this mutation is clearly catalysis-influencing. The side chain of the Ile989 amino acid residue is also turned away from the active site and is in contact (within 5 Å) with Phe 1109, Leu1138 and Met1142. Substitution of highly hydrophobic Ile for the Thr containing hydroxyl group may shift the position of this residue and influence ACE catalysis indirectly. Moreover, the different nature of the substitutions of Val for Met in the 992 position and Val for the more hydrophilic Thr in the 989 position may contribute to the differential effects of these substitutions on ACE activity.

The situation with mutation M118T located inside the N domain globule seems equivocal, as the data for two patients with this mutation did not correlate with each other (Fig 3A and 3B).

Finally, it is worth noting mutation S631C did not affect ACE activity with HHL, while it surprisingly increased ACE activity with ZPHL, as well as the ZPHL/HHL ratio (Fig 3C). This mutation is located inside the C domain of ACE but close to the N domain (Fig 1) and likely can induce conformational changes in the N domain to increase its activity towards ZPHL.

In any case, mutations leading to decreased ACE activity, especially towards ZPHL, could be risk factors for late-onset AD.

### Conformational changes in ACE molecule by ACE mutations

Further information about the effects of some mutations on various ACE properties can be obtained by an analysis of mAbs binding to different epitopes on the surface of the ACE globule. We therefore tested the binding of mutant ACEs to mAbs which contained the mutated residues in the regions of their epitopes. The newly identified transport-deficient mutation C734Y on the surface of the C domain dramatically increased ACE binding by mAb 3F10 (more than two-fold), which therefore could serve as a marker for this mutation (Fig 3D).

The binding of ACE mutant T887M by mAb 8H1 was significantly decreased, confirming a previous finding [14]. However, ACE from the blood of patient VKK056 containing two mutations, T887M in the C domain and Y215C in the N domain, exhibited increased binding by mAb 8H1 (Fig 3D). This result may indicate a remarkable influence of the Y215C mutation in the N domain on the whole ACE structure.

The binding of mutants Y992M and Y997M by mAb 2H1, as well as the binding of mutant I989T by mAb 3F10, was not altered, indicating that these mutations inside the C domain globule can influence ACE activity but not affect the topology of the surface.

The binding of ACE mutant M118T from the blood of both carriers of this mutation by mAb 2D1 was dramatically increased (Fig 3D), and the binding of ACE mutant G409S by mAbs 6A12 (Fig 3D) also changed, as expected, based on epitope 6A12 localization–Fig 1 and [31]. The binding of ACE mutant N1007K was weakly but significantly decreased by mAb 8H1 (Fig 3D). Therefore, these mAbs could be considered as markers for the corresponding ACE mutations.

Limitations of this study include the extremely small volume of plasma samples from newborns (100 ul), which preclude testing of many additional important functional and clinically relevant characteristics of mutant ACEs, such as binding of ACE inhibitors, hydrolysis of natural substrates for ACE (AI, bradykinin, Ac-SDKP) and, especially hydrolysis of beta-amyloid peptide Aβ1–42.

## Conclusions

1. Precipitation of ACE from EDTA-plasma samples by strong mAb 9B9 combined with subsequent measurement of ACE activity with ZPHL (part of the ACE phenotyping approach [16, 28–30, 32]) allowed us to estimate quantitatively the effect of different ACE mutations on blood ACE levels, as well as identify patients with decreased ACE levels (carriers of ACE mutations M118T, C734Y, V992M and V997M) who may be at risk for late-onset AD.

2. Combinations of different mAbs to ACE from our collection [13, 14] with different substrates allowed us to reveal a putative transport-deficient mutation, C734Y, and to detect carriers of ACE mutations in the region of active centers with both decreased and increased catalytic activity of the N and C domain active centers (M118T, I989T and V997M).

3. Partial ACE phenotyping using only EDTA-plasma (without the possibility to measure blood ACE activity in solution from a given individual) but using a set of mAbs to different epitopes on ACE molecule allowed us to suggest several markers for the tested ACE mutations. Especially, it is important to define mAb 3F10 as a marker for the transport-deficient ACE mutation C734Y.

4. We plan to organize limited clinical trials in carriers of some transport-deficient ACE mutations using therapeutic cocktails of chemical and pharmacological chaperones and proteasome inhibitors to rescue impaired traffic of mutant ACEs to the cell surface. Such an approach was successful for an *in vitro* model of the rare transport-deficient ACE mutation (Q1069R) in the past [10]. To estimate the effects of such therapy in clinical trials, it is necessary to quantify the probable increase of mutant ACE level in the blood of patients with already decreased blood ACE levels. We now have established a marker (3F10/9B9 ratio) for the transport-deficient ACE mutation C734Y found in this study.

5. Blood ACE phenotyping of carriers of 11 ACE mutations performed in this study allowed us to prepare an updated version of a Table with 1246 existing ACE mutations in which blood ACE levels were estimated or quantified in the carriers of 73 ACE mutations (S1 Table in S1 File). Excerpts from this Table showing only ACE mutations for which blood ACE levels were estimated or measured (S2 Table in S1 File) convincingly indicate that blood ACE levels were significantly decreased in a significant number of patients with damaging ACE mutations and Alzheimer’s disease.

## Supporting information

S1 File (PDF)
